# Blue carbon potential of coastal wetland restoration varies with inundation and rainfall

**DOI:** 10.1038/s41598-019-40763-8

**Published:** 2019-03-13

**Authors:** Karita Negandhi, Grant Edwards, Jeffrey J. Kelleway, Dean Howard, David Safari, Neil Saintilan

**Affiliations:** 1 Office of Environment and Heritage, New South Wales, Australia; 2 Department of Environmental Sciences, Macquarie University, New South Wales, Australia; 3 Department of Environmental, Earth and Atmospheric Sciences, University of Massachusetts Lowell, Massachusetts, United States

## Abstract

There is a growing interest in how the management of ‘blue carbon’ sequestered by coastal wetlands can influence global greenhouse gas (GHG) budgets. A promising intervention is through restoring tidal exchange to impounded coastal wetlands for reduced methane (CH_4_) emissions. We monitored an impounded wetland’s GHG flux (CO_2_ and CH_4_) prior to and following tidal reinstatement. We found that biogeochemical responses varied across an elevation gradient. The low elevation zone experienced a greater increase in water level and an associated greater marine transition in the sediment microbial community (16 S rRNA) than the high elevation zone. The low elevation zone’s GHG emissions had a reduced sustained global warming potential of 264 g m^−2^ yr^−1^ CO_2_-e over 100 years, and it increased to 351 g m^−2^ yr^−1^ with the removal of extreme rain events. However, emission benefits were achieved through a reduction in CO_2_ emissions, not CH_4_ emissions. Overall, the wetland shifted from a prior CH_4_ sink (−0.07 to −1.74 g C m^−2^ yr^−1^) to a variable sink or source depending on the elevation site and rainfall. This highlights the need to consider a wetland’s initial GHG emissions, elevation and future rainfall trends when assessing the efficacy of tidal reinstatement for GHG emission control.

## Introduction

For centuries humans have modified the hydrological characteristics of low-lying coastal environments to promote agricultural and aquaculture development, to mitigate flood risk, to control insect infestation or as an artefact of transport corridors^1,2^. This has largely been achieved through the imposition of levee banks restricting tidal exchange, leaving wetlands to be either drained or freshened. Tidal wetland loss has accelerated through the second half of the 20^th^ century^3^, with largescale coastal reclamation occurring in mangrove forests of SE Asia^4^, coastal wetlands of China^5^, coastal floodplains in eastern Australia^6^, and North American saltmarshes^7,8^. Such modifications have resulted in up to a 50% reduction of tidal wetlands worldwide since 1900^9^.

The isolation of tidal wetlands alters the biogeochemical characteristics of soils, with profound implications for greenhouse gas emissions (GHG; CO_2_ and CH_4_). Wetland drainage promotes the remineralization of organic carbon, leading to substantial CO_2_ emissions^10^ and provides opportunity for aerobic oxidation of CH_4_^11^. Reduction of tidal saltwater connections can also enhance methane (CH_4_) production^12–14^, as sea-water associated microbes can reduce CH_4_ emissions through two processes: (1) hindering production by outcompeting methanogenic archaea for electrons^15^ and (2) promoting the anaerobic oxidation of CH_4_^16^. The obstruction of tidal flow to coastal wetlands over several continents has likely created additional freshwater wetland CH_4_ sources, contributing to global warming^17^, though few tests of this assumption are documented.

The potential GHG mitigation benefits of tidal reinstatement are additive: CH_4_ emission reduction is accompanied by the restoration of “blue carbon” ecosystems, with mangrove and saltmarsh being noted for relatively high rates of carbon sequestration^18–20^. In the context of emerging carbon trading markets^21^ and national greenhouse gas inventories^14^, the prospect of tidal reinstatement appeals to managers and investors as an offset mechanism for several reasons. First, the opportunities are likely to be numerous and at a large spatial scale (thousands of hectares). Second, measurable benefits are potentially gained over short time periods (weeks to months), being mediated by rapidly responding hydrological and microbial conditions. Third, avoided emissions are permanent, in the sense that the benefit is not negated by subsequent management interventions or pertubations^8,22^.

There is currently little guidance on how to account for the emission benefits of tidal reinstatement of impounded marshes. The 2013 Supplement to the 2006 IPCC Guidelines for National GHG Inventories: Wetlands^14^ makes no reference to tidal (saline) reinstatement of impounded wetlands as a blue carbon mechanism, nor does it allow for the comparison of emission factors for impounded and natural tidal wetlands. Kroeger *et al*.^8^ provide suggested emissions factors, though these are based largely on Poffenbarger *et al*.^13^ who compared emissions across a gradient of salinities, and the emissions factors reported in Hiraishi *et al*.^14^. There is clearly insufficient experimental data on whether tidal reinstatement has the promised benefit for significant emissions reduction.

The Hunter estuary of New South Wales (NSW), Australia underwent tidal restriction in 1956, and over ensuing decades impounded formerly tidal floodplains have transitioned to brackish and freshwater wetlands^23^. More recently, several large-scale tidal reinstatements^24^ have restored over 1000 ha of coastal tidal wetland prior to the third phase of tidal reinstatement at Tomago in 2015. We took the opportunity to monitor the Phase 3 tidal reinstatement to test the following three hypotheses: (i) tidal restoration would alter microbial assemblages increasing the representation of sulfate-reducers; (ii) tidal reinstatement would transition the restored wetland from a contributor to a mitigator of radiative forcing through reduced CH_4_ emissions; and (iii) these changes would be similar across low and high intertidal elevations within the restored wetland.

## Study Approach

Monitoring equipment was installed 3.5 months before floodgates at Tomago, NSW wetland were lowered to re-introduce tidal waters (12 Nov 2015), to measure water level, salinity, conductivity, and CO_2_ and CH_4_ flux (using an Eddy co-variance tower). Equipment remained for 8.5 months after tidal reinstatement. Periods of exceptionally high rainfall occurred following tidal reinstatement, which allowed us to explore the influence of rainfall on gas flux. The Eddy covariance tower was positioned in the wetland such that its flux footprint would cover high and low elevation zones according to the wind direction. Two sampling locations below mean sea level (−12 cm Australian Height Datum (AHD) and −8 cm AHD) and two sampling locations above mean sea level (12 cm AHD and 8 cm AHD) were identified for sampling of microbial communities. All four locations were within 40 m of the Eddy co-variance tower. Rainfall data were acquired from the nearby (~12.5 km) weather station of Williamtown RAAF, NSW.

## Results and Discussion

### Hydrological changes

**Figure 1:**
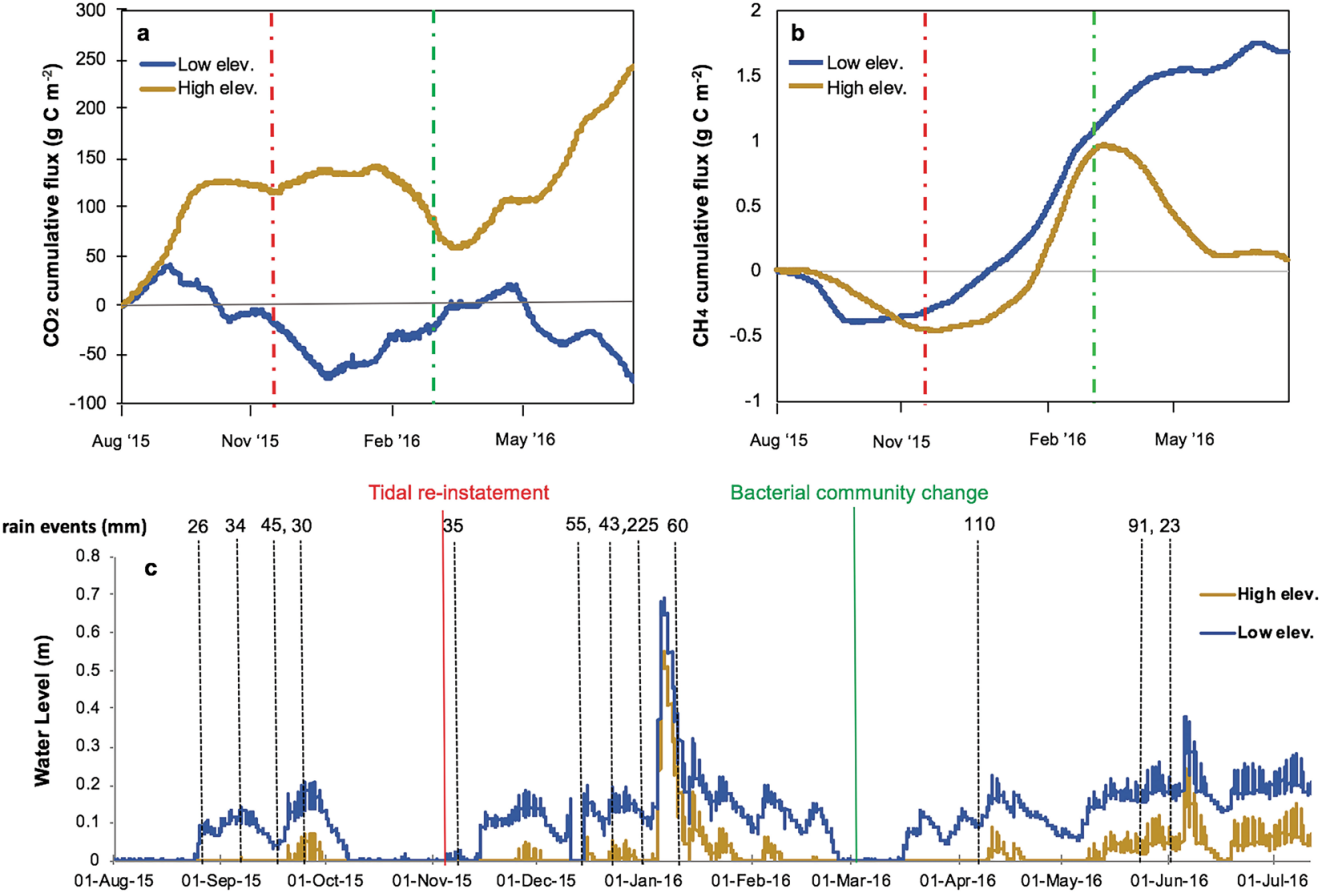
Flux lines at high and low elevation sites for (**a**) CO_2_ and (**b**) CH_4_. Flux is cumulative for each 30 minute measurement period. Vertical lines indicate when floodgates where open (red, 12 Nov 2015) and when a change in the microbial community was detected at the low elevation (green, Mar 2016). Area between these vertical lines are when extreme rainfalls occurred, which are indicated with (**c**) timeline of water level, rain events >25 mm, and tidal reinstatement for low elevation and high elevation sites.

Before reinstatement, water levels ranged from 0 to 0.69 m and 0 to 0.55 m for the low (−0.04 m AHD) and high (0.10 m AHD) elevations sampling locations respectively, with high rainfall events the primary drivers for large inundation events (Fig. 1c). Surface runoff from the hinterland likely contributed to higher water levels at low elevation. Overall, tidal reinstatement increased inundation of the wetland, with the proportion of time inundated increasing from 66% pre-reinstatement to 91% post-reinstatement for the lower elevation sampling locations and from 21% to 50% for the higher elevation sampling locations.

Within four days after tidal reinstatement a rain event of 35 mm occurred and was followed by two extreme rain periods of 123 mm and 422 mm over several days (22 to 27 Dec and 5 to 18 Jan, respectively) five weeks later. Both of these events were above the regional average rainfall for December and January of 80 and 100 mm, respectively. Two more above-average rain events also occurred in April (147 mm) and June (151 mm). These large rain events still induced spikes in water level (Fig. 1c) and were followed by a decrease in salinity (Supplementary Fig. S1).

### Microbial communities as an indicator of tidal impact

**Figure 2:**
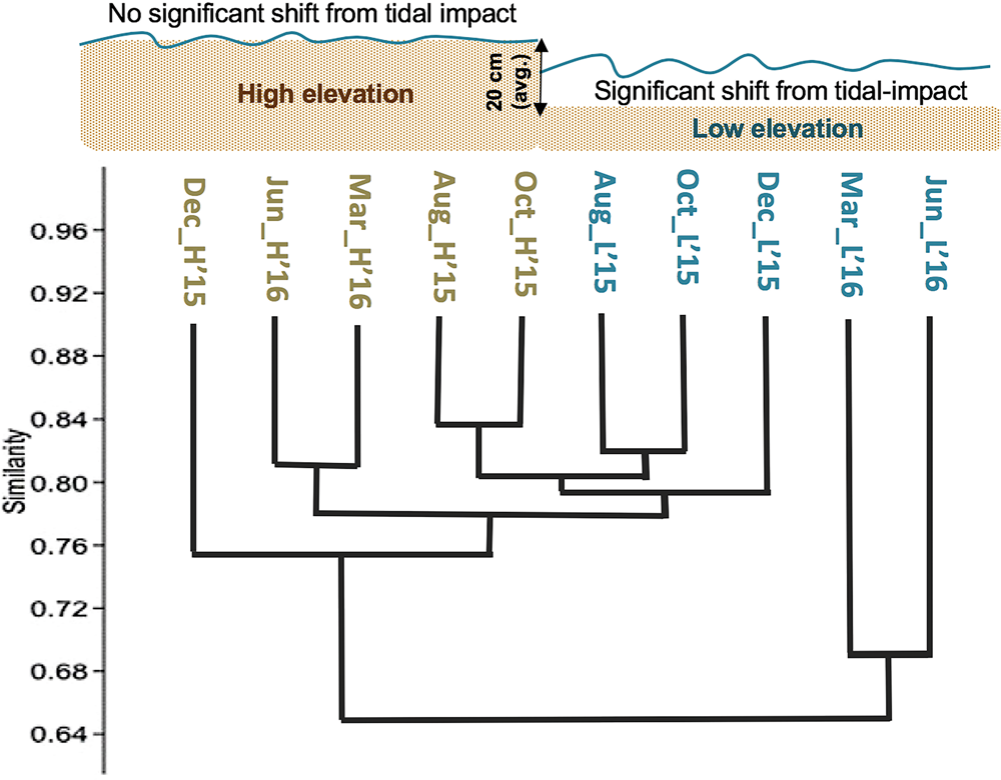
Surface sediment bacterial community dissimilarity tree (Bray-Curtis; shared OTUs at 97%). Branch lengths indicate the amount of differentiation among communities. March and June low elevation communities after tidal reinstatement have the longest branches and form their own cluster (31% dissimilar from other communities), indicating a significant change in the community occurring at the taxonomic level of Order (ANOSIM p < 0.01, R > 0.69; Supplementary Fig. S3). Each community time point represents two bacterial samples of 11,062 sequences (reads).

Microbial communities collected before and after tidal reinstatement at high and low elevation sites were used to identify temporal trends in response to tidal inundation. The more diverse surface sediment microbial communities at the low elevation (Supplementary Tables S1 and S2) site sustained a significant change after tidal reinstatement between December and March (ANOSIM p < 0.01, global R > 0.69; Supplementary Fig. S3). December samples, 25 days post-reinstatement, clustered with ‘before’ communities and also with the high elevation sites communities, which did not show a significant community change (Fig. 2). By March 2016, the ‘after’ reinstatement community was dissimilar to communities ‘before’ reinstatement. The top 3 Orders that decreased after tidal reinstatement at the low elevation sites were ones commonly associated with freshwater sediment (*Rhodospirillales* and *Rhizobiales*)^25^ and soil (*OPB35 soil grp*. *uncls*.)^26^. Together they contributed 7.4% (SIMPER) of the community dissimilarity after tidal impact. After March, the water level and salinity at the low elevation site was consistently >0.1 m and >12.7 ppt respectively, indicating a more sustained saltwater inundation. The bacterial community also shifted to one more representative of a marine environment. The top two Orders that increased were *Desulfobacterales* and *Clostridales*, contributing to 7% of the community shift after flooding (Supplementary Fig. S2). *Desulfobacterales* are anaerobic and often found in saline environments^27^. Moreover, together with the presence of *Clostridales*, they have the functional capability to take up carbonate and fix CO_2_^28^. The archaeal community was dominated by *Woesearchaeota DHVEG-6* and *Archaea unclassified* (Supplementary Fig. S5) at both elevations, yet the low elevation area experienced a 15% community change after tidal reinstatement, which is attributed to three Genera commonly associated with higher salinities (*Halarchaeum*, *Halobacteriales unclass*. *and Marine Benthic Group-B unclass*.; Supplementary Fig. S2).

### GHG patterns before and after tidal reinstatement

Natural and managed wetlands are known to have different GHG emissions^29,30^. The observation of an impounded wetland as a CH_4_ sink (−0.07 to −1.74 g C m^−2^ y^−1^) is unusual (before tidal reinstatement, Table 1), but can be explained in terms of salinity. Before tidal reinstatement, the salinity average was slightly <18 ppt (17.4 avg) and ranged from 16.7 to 18.3, with the presence of a sulfuric soil layer at ~0.3 m deep^31^ likely contributing to inhibiting of CH_4_ production. Water level increases from rain events did increase the CH_4_ flux, but not enough to impede the presence of a CH_4_ sink (Fig. 1b, Aug – 12 Nov). Another eastern Australian coastal wetland (~800 km North), subject to substantial freshwater input was also a reported CH_4_ sink (−3.4 mg C m^−2^ y^−1^) when the groundwater table was high (−252 mm)^32^. These hitherto overlooked ecosystem sinks are not insignificant compared to the global average soil sink strength for CH_4_ (0.003–20.7 g C m^−2^ yr^−1^; ref.^33^).

**Table 1:** Carbon dioxide and CH_4_ emissions for both high and low elevation sites before and after tidal reinstatement with associated standard errors.

	CO_2_ g C m^−2^ yr^−1^	CH_4_ g C m^−2^ yr^-1^	CO_2_ g C m^−2^ yr^-1^	CH_4_ g C m^−2^ yr^-1^
	High Elevation		Low Elevation	
**All Events**				
Before (Aug–Nov)	420 ± 22	−1.74 ± 0.04	−57 ± 31	−0.07 ± 0.08
After (Nov–Jul)	191 ± 23	0.82 ± 0.07	−94 ± 32	2.40 ± 0.08
After (Mar–Jul)	430 ± 28	−1.92 ± 0.08	−152 ± 29	1.03 ± 0.12
**No Rain**				
Before (Aug–Nov)	420 ± 23	−1.67 ± 0.05	−63 ± 32	−0.12 ± 0.08
After (Nov–Jul)	350 ± 24	−0.65 ± 0.07	−180 ± 29	1.54 ± 0.09
After (Mar–Jul)	522 ± 28	−2.59 ± 0.09	−217 ± 27	0.67 ± 0.01

Emissions were calculated for immediately after tidal inundation (Nov–Jul), when a change in the surface bacterial community was detected (Mar–Jul), and with the removal of rain event impacts (Supplementary Fig. S1) to see the true influence of tidal reinstatement on GHG emissions.

**Figure 3:**
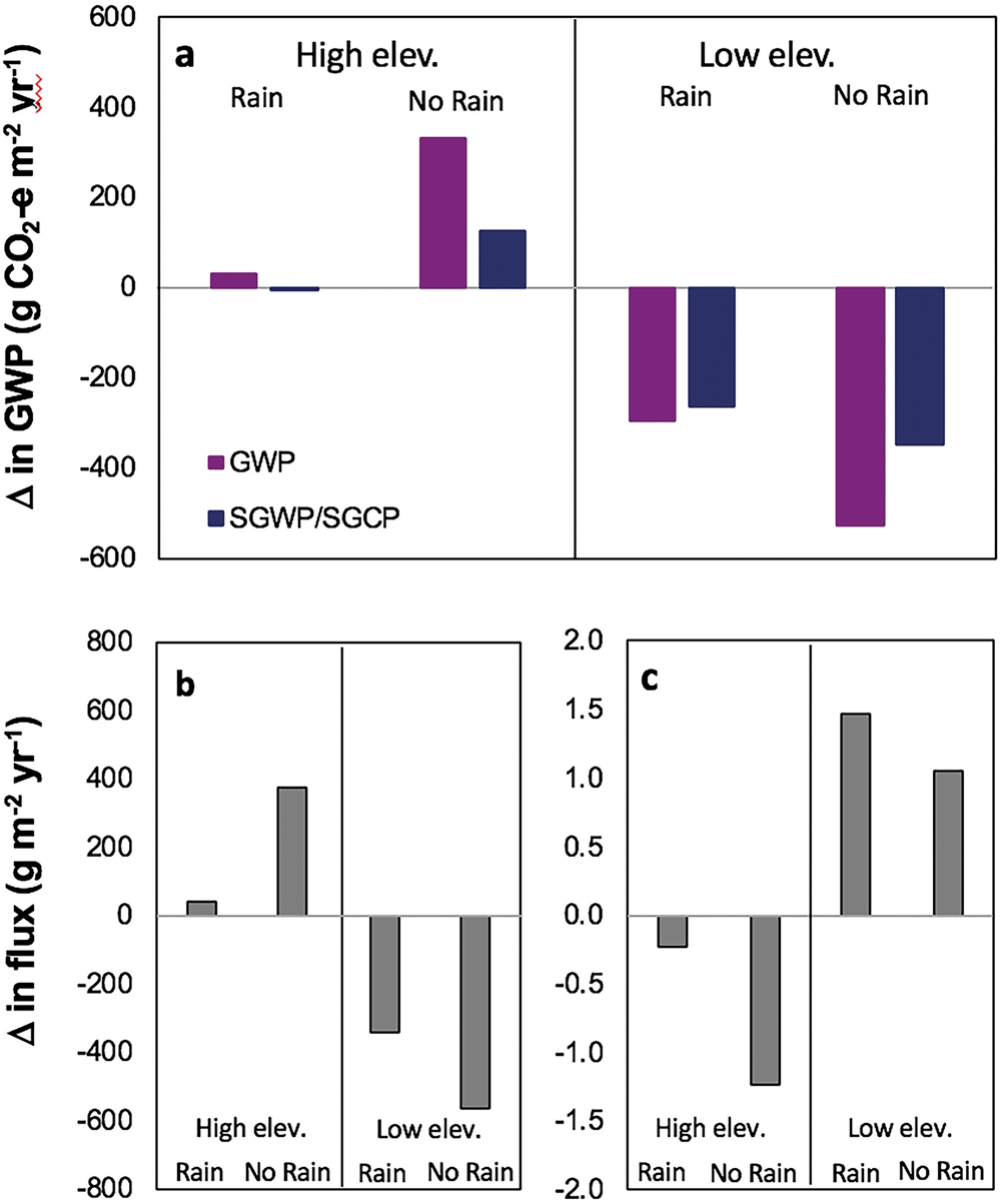
The low elevation zone had a reduced carbon flux in CO_2_-equivalents after tidal reinstatement, especially with a subset of rain events removed (Supplementary Fig. S1). Despite CH_4_’s higher radiative forcing than CO_2_, the small change in its flux after inundation compared to CO_2_ means GWP follows the CO_2_ trend. (**a)** Change in CO_2_-equivalents between before (Aug–Nov) and after (Mar–Jul) tidal reinstatement for high and low elevation sites using global warming potential (GWP*; violet) and a sustained global warming/cooling potential (SGWP/SGCP**; black). Both are for over 100 yrs and including rain events. Change in (**b**) CO_2_ and (**c**) CH_4_ flux for before (Aug–12 Nov) and after (Mar–Jul) tidal impact. *CH_4_ expressed in CO_2_ equivalent as IPCC’s GWP for 100 yrs (x34; this factor includes climate carbon feedback, such as CH_4_ oxidation to CO_2_^34^). **CH_4_ expressed in CO_2_ equivalents as sustained global warming/cooling potential for 100 yrs (x45 for warming and x203 for cooling). Here the sustained gas warming (positive CH_4_ flux) and the sustained gas cooling (negative CH_4_ flux for uptake/sink) are representative of a flux that persists over time rather than a one-time pulse^35^.

The increase in salinity in late February and shift in bacterial community detected at the low elevation site in March indicates that the above average rainfalls during Dec – Jan may have impeded the initial saltwater introduction from tidal reinstatement in November. Using March as the starting point for when GHG emissions reflect tidal influence, the low elevation zones CO_2_ flux decreased by 95 g C m^−2^ yr^−1^, which is similar to the CO_2_ tier 1 emission factor benefits for restored saltmarsh that has been drained for >30 years (91 g C m^−2^ yr^−1^;^8^). The low elevation zones increased CH_4_ flux of 0.96 g C m^−2^ yr^−1^ (Table 1). While the average CH_4_ emission was positive from March – July, a net GHG reduction was still achieved ranging from −296 to −264 g m^−2^ yr^−1^ of CO_2_-e (CO_2_ equivalents) over 100 yrs (Fig. 3a). This net reduction range was seen through the use of a single pulse metric, global warming potential (GWP)^34^, and also with using a sustained warming/cooling (SGWP/SGCP)^35^. Within the same time period, the high elevation area ranged from a net increase in CO_2_ equivalents using GWP (27 g m^−2^ yr^−1^) to a slight reduction using SGWP/SGCP (−9 g m^−2^ yr^−1^). Even with the higher radiative forcing of CH_4_, the carbon emissions are dominated by CO_2_ emissions for both elevation categories (Fig. 3b,c). These results underscore the need to measure both CO_2_ and CH_4_ in characterising the effects of tidal reinstatement on GHG flux.

Importantly, the outcomes were quite different between the adjacent higher and low elevation wetland zones. After tidal reinstatement, the low elevation zone had a reduced atmospheric GWP compared to the high elevation increasing its GWP (Fig. 3a). These emission reductions were achieved unexpectedly through a reduction in CO_2_ and in spite of an increase in CH_4_. This ran counter to the expected blue carbon emission reduction mechanism of neutral benefit in CO_2_ and reduction in CH_4_^8^. We attribute this to (i) a low to negative CH_4_ emission prior to reinstatement associated with higher than expected salinity and (ii) the significance of rainfall influencing GHG flux post-reinstatement, particularly at the higher elevation site.

### Rain Influence

Spikes in CH_4_ emission from both the low elevation and high elevation zones were observed in relation to a succession of high rainfall events. The late December to January events, shortly following tidal reinstatement, provide an example (Fig. 1b,c). Together these events decreased the salinity to ~7 ppt (Supplementary Fig. S1), well below the 18 ppt threshold for decreased CH_4_ production proposed by Poffenbarger *et al*.^13^. This increased CH_4_ production is sustained across both elevation zones until March when the salinity started increasing (Supplementary Fig. S1). A decrease or plateau in CH_4_ cumulative flux also occurred in March and was sustained through July for both zones. This coincided with an increase in inundation time at the high elevation sampling site from 0% in March to 94% in June and 100% in July. The low elevation site was inundated 58% in March to 100% of the time from April to July.

**Figure 4:**
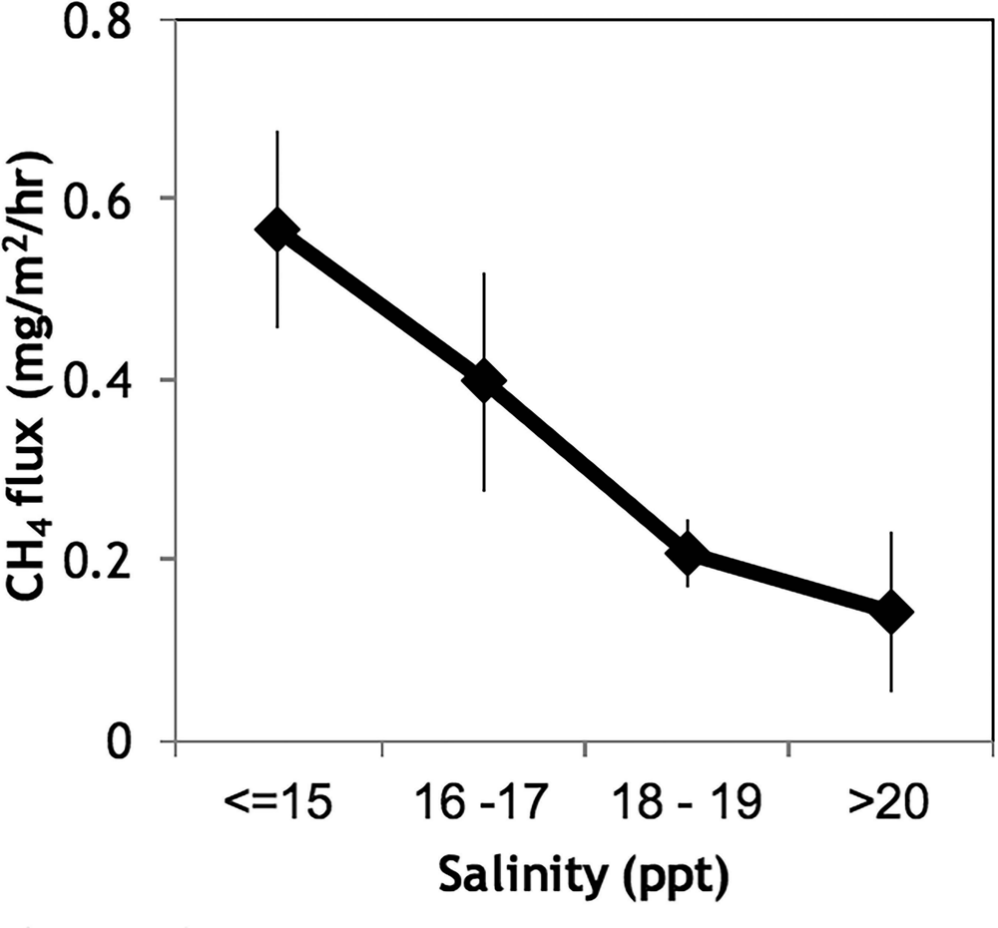
Decrease in CH_4_ flux with increase in salinity from the low elevation site. Error bars show variability in flux within salinity ranges. When salinity is ≤17.43 ppt, the CH_4_ flux is significantly higher (p = 0.047, d = 0.1579; kolomogorov-Smirnov).

There is evidence that the presence of a more sustained tidal inundation time in the low elevation zone acted to buffer the impact of rainwater decreasing soil salinity. During a 3-day rain event (DOY 156–158; 114 mm; Supplementary Fig. S1), the salinity increased to 18 ppt reflecting a controlling influence of tidal ponding on salinity and CH_4_ flux. Indeed, when salinity levels were <17.45 ppt, CH_4_ flux was significantly higher (p = 0.047; Fig. 4). Removing time periods where rainfall decreased salinity and increased fluxes (Supplementary Fig. S1), the net CH_4_ flux decreased for both elevation zones after flooding (Table 1). Tidal reinstatement does not therefore prevent the freshening of upper intertidal habitat during high rainfall periods, and attendant CH_4_ production. However, the presence of surface flooding by tidal inundation decreased the duration of exposure to rainwater induced CH_4_ emission.

Freshwater inputs from the large rain events corresponded to opposing CO_2_ trends for the two elevation zones. They occurred in relation to reduced CO_2_ emissions at the high elevation zone and increased CO_2_ emissions at the low elevation zone. At the low elevation zone, the decrease in CO_2_ flux (Fig. 1a), was possibly an artefact of inundation (Table 1; after all events) and the increased presence of *Desulfobacterales* and *Clostridales* following reinstatement. For both CO_2_ and CH_4_, the influence of rain events was more pronounced for the high elevation zone, as less tidal influence left fresh rainwater to dominate inundation periods (Fig. 1c). As previously seen^36^, CO_2_ flux responses to rain were more rapid than CH_4,_ which exhibited a time lag (Supplementary Fig. S1) during soil rewetting. Rain events would no longer be ‘rewetting’ the soil, in the low elevation zone, once it transitioned to sustained inundation (58 to 100% inundation time from Mar – Jul), and hence no CO_2_ flux reaction was seen to rain events after March.

Excluding these rain events, the low elevation zone transformed to a CO_2_ sink (−217 g C m^−2^ yr^−1^) and lower CH_4_ source (0.67 g C m^−2^ yr^−1^), while the high elevation zone was a CO_2_ source (522 g C m^−2^ yr^−1^) and CH_4_ sink (−2.59 g C m^−2^ yr^−1^) over the same period (Table 1). Less inundation time and greater exposure of surface sediments to the atmosphere in the high elevation zones likely supports the stronger CH_4_ uptake rates. With the removal of rain events, the low elevations CO_2_ sink strength is >2x as strong as that reported for restored saltmarshes (−91 g C m^−2^ yr^−1^;^14^). The ability of the low elevation zone, under anoxic conditions, to mediate CH_4_ emissions and decrease emissions resulted in reduced carbon emissions including rain events or excluding rain events (Fig. 3).

## Conclusions

Our results show that a rapid shift to a reduced GHG ecosystem can occur within three months after tidal reinstatement, mediated by a relatively rapid transition of the microbial community to become more representative of marine conditions. We observed fundamental differences in the greenhouse gas dynamics between elevation zones within a single wetland, with the low elevation zone experiencing prolonged tidal inundation (Fig. 1c), greater microbial community change (Fig. 2), and a lower carbon GWP/SGWP (Fig. 3). The results provide a unique insight into the impact tidal reinstatement can have in promoting a transition to a blue carbon ecosystem, though we acknowledge the lack of true experimentation control.

The GHG benefits of tidal reinstatement are mediated by rainfall inundation. Here large rain events over the summer impeded the influence of tidal reinstatement on microbial assemblages until our autumn sampling. Furthermore, these soils have been classified as potential acid sulfate soils^31^ and did harbour sulfate reducing bacteria before inundation. The combined presence of sulfate reducers (which outcompete methanogens for electrons required for CH_4_ production^15^) and drier soils facilitating aerobic oxidation of CH_4_^11^ can help explain the CH_4_ uptake prior to tidal reinstatement. The presence of sulfate-reducing bacteria could have also aided in a more rapid community transition once reinstatement occurred. The Family that increased the most after tidal reinstatement was *Desulfobacteraceae*, which includes taxa that are sulfate-reducing bacterial partners of anaerobic methanotrophic archaea carrying out anaerobic oxidation of CH_4_^37^. Therefore, these minerogenic soils provide an ideal coastal environment where blue carbon storage can occur with tidal reinstatement. The caveat is that in lowland floodplain environments with a prior Holocene history of seawater inundation, post-impoundment conditions may still be conducive to anaerobic CH_4_ oxidation, rendering the net GHG benefits primarily through CO_2_ reduction. With CO_2_ and CH_4_ having substantially different radiative forcings^38^, the GHG benefits of blue carbon coastal ecosystems will likely vary spatially.

Net GHG emissions were reduced in the tidally restored low elevation wetland zone but not in an adjacent higher elevation zone, which experienced lower levels of tidal inundation and greater exposure to rainfall ponding. This highlights a critical need for further experimental data on the relationships between elevation, hydrology, climate and GHG dynamics. It is possible, for example, that the observed die-off of grasses (*P*. *vaginatum*) and sedges (*B*. *caldwellii*) following the conversion of the low elevation site to more permanent flooding decreased autotrophic respiration. The capacity of high to extreme rainfall events and ensuing brackish groundwater fluxes to interrupt the methane sink potential of low elevation zones highlights the need to consider rainfall trends, including shifts in rainfall intensity under climate change^39^, when assessing the efficacy of tidal reinstatement as an emission control mechanism.

## Methods

### Site

The field experiment was carried out at Tomago wetland (−32.9487°; 151.8347°) within the Hunter estuary of NSW, Australia. Prior to tidal reinstatement, the brackish grassland was dominated by *Paspalum vaginatum*. The high elevation site also included the remnant saltmarsh species *Sporobolus virginicus*, *Triglochin striatum* and *Juncus kraussii*. Comparatively, the low elevation site was intermixed with *Bolboschoenus caldwellii*. Following tidal reinstatement, lower elevation area transitioned to open water and/or mudflat, and the presence of *P*. *vaginatum* decreased across the wetland. Mature Swamp Oak Floodplain Forest bordered north, east and south of the wetland and a narrow strip of tall (~2 m) *Phragmites australis* reedswamp bordered the west of the wetland for the duration of the monitoring period.

### Sampling design

Before floodgates were lowered to reinstate tidal flow on 12 November 2015, monitoring equipment was installed. This included water level (April 2015, HOBO® U20L), salinity (15 Oct 2015, 36 cm depth, HOBO® U24 conductivity and temperature logger), and an Eddy co-variance tower (4 Aug 2015, LI-COR®). The Eddy covariance tower was positioned in the middle of the wetland allowing its flux footprint would pass over both zones according to the wind direction. Two sampling locations below mean sea level (−12 cm AHD) and −8 cm AHD, and two sampling locations above mean sea level (8 cm AHD and 12 cm AHD) were identified for sampling of microbial communities. All four locations were within 40 m of the Eddy co-variance tower.

Rainfall data were acquired from the nearby (~12.5 km) weather station of Williamtown RAAF (Australian Bureau of Meteorology, www.bom.gov.au). All monitoring equipment was left for 7.5 months following tidal reinstatement. Rainfall events >25 mm (Fig. 1) and decrease in salinity were used to identify times freshwater input from rainfall impacted carbon flux (Supplementary Fig. S1). Removal of these times (shaded grey in Supplementary Fig. S1) represent ‘No Rain’ data. Microbial samples were collected five times throughout the study. At each sampling time point, four samples were collected with two replicates taken at separate locations within each elevation site (details below).

### Water level

A HOBO U20L water level loggers (Onset Computer Corporation, Massachusetts, USA) were deployed on April, 2015 – adjacent to a low elevation sampling site (elevation of logger = −0.04 m AHD). Pressure measurements were recorded at 15 minute intervals, and water depth estimated by correcting for barometric pressure data from a nearby weather gauge (Williamtown RAAF). Water levels for the high elevation sampling site (mean elevation of 0.10 m AHD) were modelled based on the vertical difference between this mean high elevation and that of the low elevation water logger (i.e. +16 cm elevation).

### Eddy co-covariance flux

A complete *Li-Cor* (Li-Cor Biosciences), Eddy Covariance system consisting of the Li-7500 and Li-7700 open path fast response sensors for CO_2,_ H_2_O, and CH_4,_ coupled with a Gill Windmaster 3D sonic anemometer and the Li-7550 with SmartFlux for real-time data processing was deployed at the Tomago site. Samples were collected at 20 Hz at a tower height of 3.45 meters above the surface. Quality control flags were calculated for each averaging period using the scheme of Foken *et al*.^40^ and fluxes given a 2 flag were discarded from further analyses. Calculation of eddy covariance fluxes and quality control was undertaken using EddyPro 5.2.1 (Li-Cor Biosciences) and further calculations/statistical analyses were performed using Matlab 2012b (The Mathworks Inc., Natick, MA, USA) and R version 3.3.3.

### Gap filling for cumulative flux

Determination of flux footprint regions was achieved using the two-dimensional footprint method of Klujn *et al*.^41^. The total spatial extent of the footprint was set as the region accounting for 80% of the flux intensity calculated by this method. Two impact regions were defined for sectors between 135° and 280° (low elevation, south and south-west in relation to the Tower) and for >280° or <135° (high elevation, north and north-east in relation to the Tower). Flux footprints were then assigned to a region only if >80% of the total flux intensity within the defined total spatial extent was found to originate within the appropriate sector. For each flux species within each impact region, gaps in the data were filled according to time of day by averaging temporally local observed values. An initial two-week period was centred about each unknown flux value and the estimated flux was calculated as the mean of all observed flux values, for the same 1.5 hour of day, within this two-week period. A minimum of 5 observed values was required for each gap fill calculation. If less observed flux values were found within the initial two-week period then this period was incrementally extended by two days (one before and one after) until the minimum number of observed flux values was acquired. Cumulative and average fluxes for both impact sites across the study period were then calculated from all observed and calculated flux values.

### Microbial samples

Surface soil and sediment samples were collected in Aug 2015, Oct 2015, Dec 2015, Mar 2016 and Jun 2016 between 12:00 and 2:00 pm. At each sampling time point, four samples were collected with two replicates taken at separate locations within each of the ~1.0 ha elevation sites. Care was taken to avoid sampling in soils disturbed by foot traffic or prior sampling. A sterile 15 ml falcon tube was used as mini corer to a depth of 2.3 cm. This 3 ml of soil or sediment was placed on ice and transferred to an −80 °C freezer within 2 hrs of collection. Samples were homogenized (mixed within tube after thawed) before transferring 0.25 g to use for DNA extraction following instructions of the MO BIO Kit (PowerSoil DNA Isolation Kit #12888). Extracted DNA was diluted to concentrations of 5.3 to 16.9 ng/µL (nan-drop spectrophotometer) and amplification was performed following the Earth Microbiome Project^42^. Briefly, a PCR reaction mixture of PCR grade H_2_O (13 µL), MasterMix (10 µL, 5 Prime HotMaster Mix), template DNA (1 µL), and 10 µM of each MiSeq primer (515 F and 806 R, both 16 S rRNA primers; 0.5 µL). These primers have recently been shown to have a bias against *Crenarchaeota*/*Thaumarchaeota*^43^. Amplification cycles included denaturing at 94 °C for 3 minutes, 35 cycles of denaturing at 94 °C for 45 s, annealing at 50 °C for 1 min, extensions at 72 °C for 1 min 30 s, and a final extension at 72 °C for 10 min. Most samples amplified using 1 µL of template DNA, except one sample in the high and low impact site from October were only capable of amplification at 1/10 µL. PCR products were purified, pooled in equal volumes (SequalPrep Normalization plates, ThermoFisher), and sequenced (MiSeq) at UNSW’s Ramaciotti Centre for Genomics. Raw reads were submitted to NCBI Sequence Read Archive reference number SRP131614.

Resulting paired-end reads were subjected to a MiSeq pre-processing, quality control, and taxonomic analyses^44^. Paired reads were aligned, with base pair quality scores >25 given preference. Matched paired reads were then removed if they were longer >275 bp, and any ambiguous base pairs (N’s). Reads were aligned using Mothur (v.1.38.0) against SILVA database (V123). Misaligned reads outside the targeted area, chimeras, and non-targeted assignments (chloroplast, mitochondria, and eukaryotes) were removed. Mothur OTUs were assigned (97% similarity) and samples were randomly resampled to 11,062 reads per sample for downstream analysis. Diversity measures were performed in Mothur. Bray Curtis similarity tree, NMDS plot, ANOSIM and SIMPER were performed in PAST (3.16).

## Supplementary information

Blue carbon potential of coastal wetland restoration varies with inundation and rainfall
