# Supplementary material for: Blue carbon potential of coastal wetland restoration varies with inundation and rainfall
Source: Sci Rep. 2019 Mar 13;9:4368. doi: 10.1038/s41598-019-40763-8 (PMC6416304; doi:10.1038/s41598-019-40763-8)
Supplement: Supplementary file 1 — Blue carbon potential of coastal wetland restoration varies with inundation and rainfall [file 41598_2019_40763_MOESM1_ESM.doc]

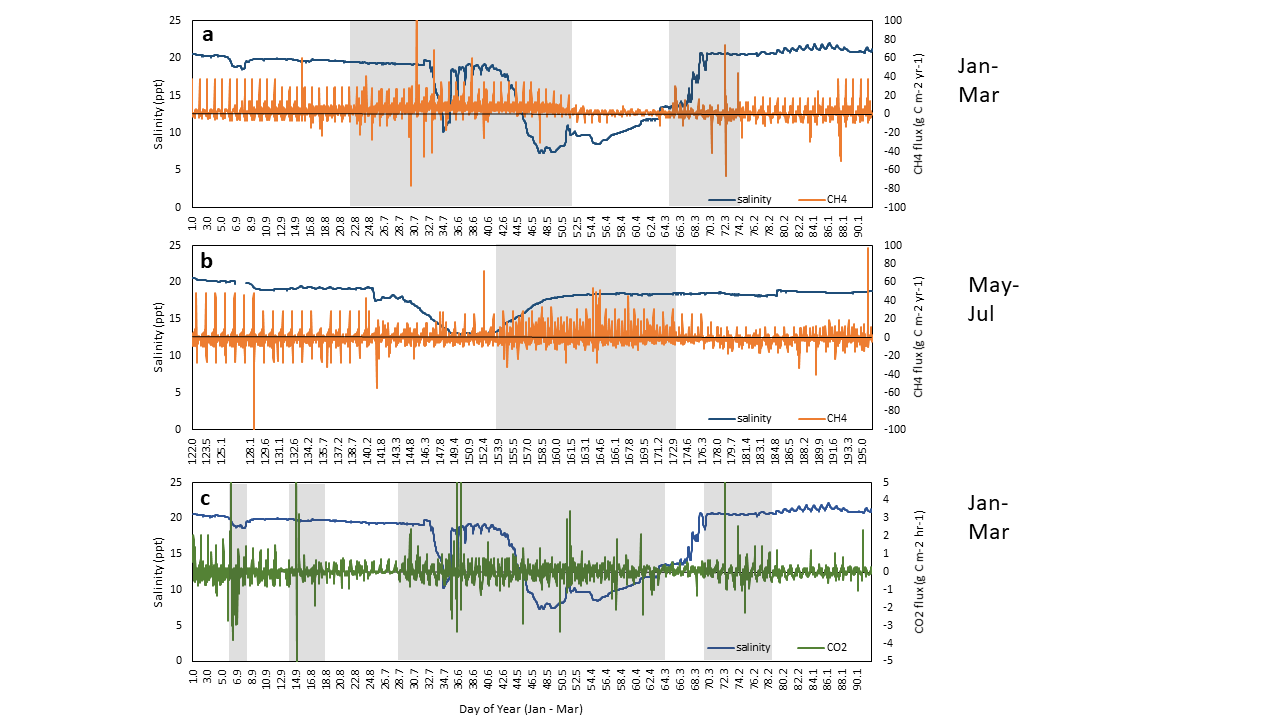


**Supplementary Figure S1**: The highlighted grey sections are when reactions in CH_4_ flux **a)** Jan ‘16 – Mar ‘16 and **b)** May ‘16 – Jul ‘16 and CO_2_ flux **c**) Jan ‘16 – Mar ‘16 due to rainfall and decreased salinity in the low elevation zone were observed. These grey sections were removed from the data set to represent the ‘No Rain’ flux values. Controlling flux factors for CH_4_ and CO_2_ are likely different. For instance, a more pronounced time lag in CH_4_ flux reaction to rainfall is seen for the rainfall event in May ’16 (DOY 141; c) whereas no reaction in CO_2_ flux was seen. Therefore, the observed reaction to rain and salinity were examined separately.


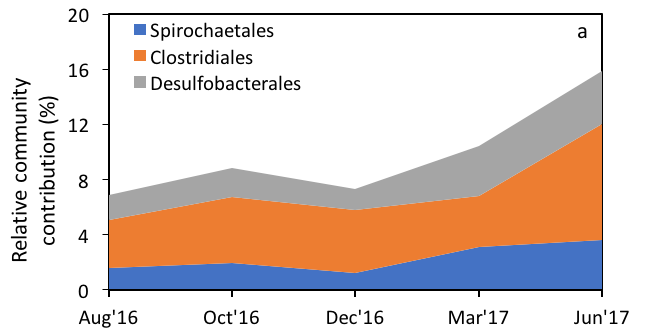


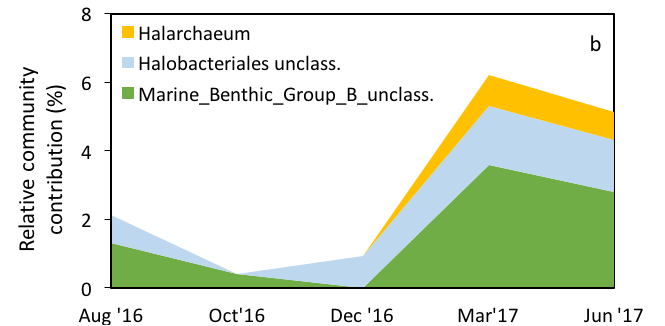


**Supplementary Figure S2**: Top 3 contributors to the significant microbial community change that occurred after tidal reinstatement in the low elevation area **a**) bacterial Orders contributing to 9.3% of the bacterial community shift, **b**) archael Genera contributing to a 15% community change. Contributions to the community shift were identified by SIMPER analysis, with each time point representeing an average of two samples within the low elevation area. The taxonomic levels of Order for bacterial communities was based on the highest level a significant difference occurred at.

Before - High

Before - Low

After - High

After - Low

**
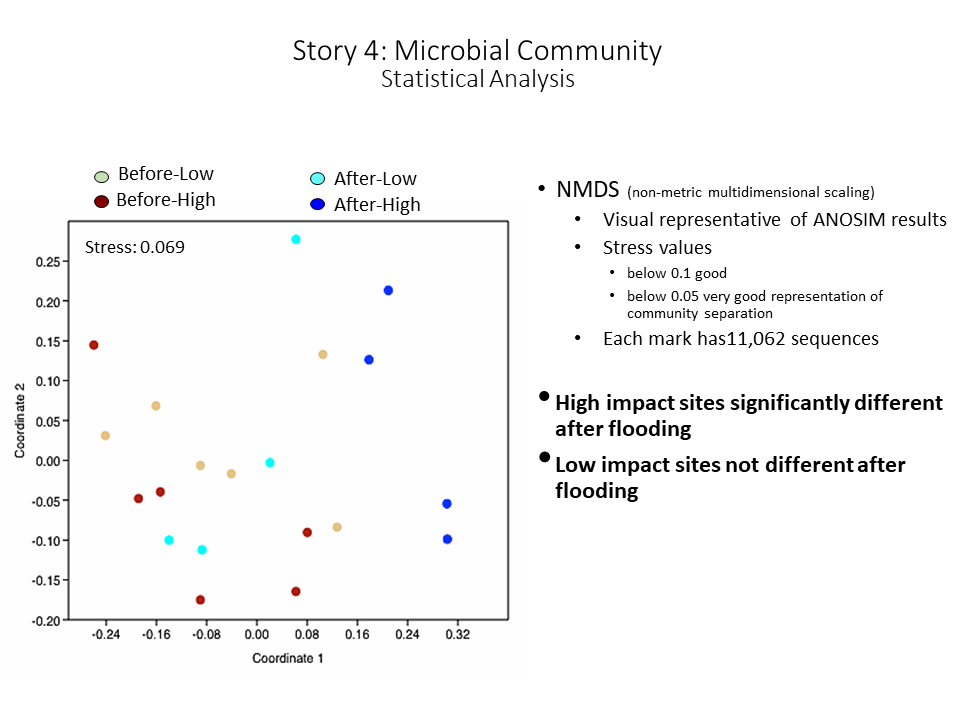
**

**Supplementary Figure S3:** Non-metric multidimensional scaling (NMDs) plot of a significant change between before and after tidal re-instatement sediment bacterial communities, at the taxonomical level of Order, for the low elevation site (ANOSIM p < 0.01, r^2^ > 0.69) but not the high elevation site (ANOSIM p > 0.06, r^2^ < 0.48) after tidal reinstatement. Order was the highest taxonomic level that significant difference was found at.


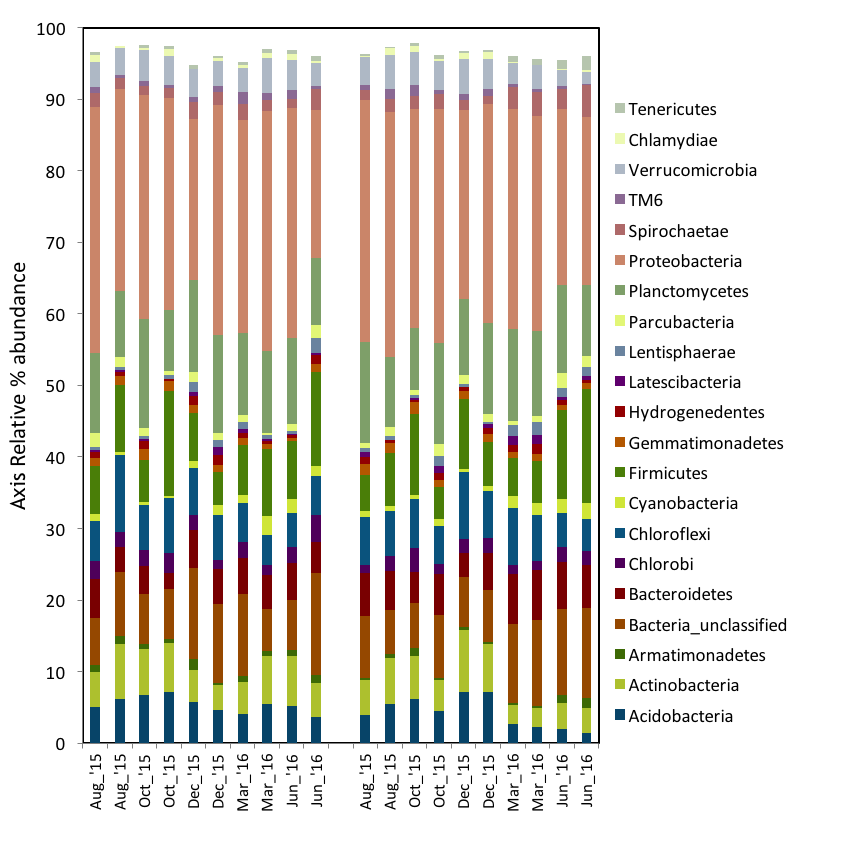


**Supplementary Figure S4**: Bacterial community relative composition of abundant Phyla (≥ 1%) of surface sediment for the high elevation area (left) and low elevation area (right).


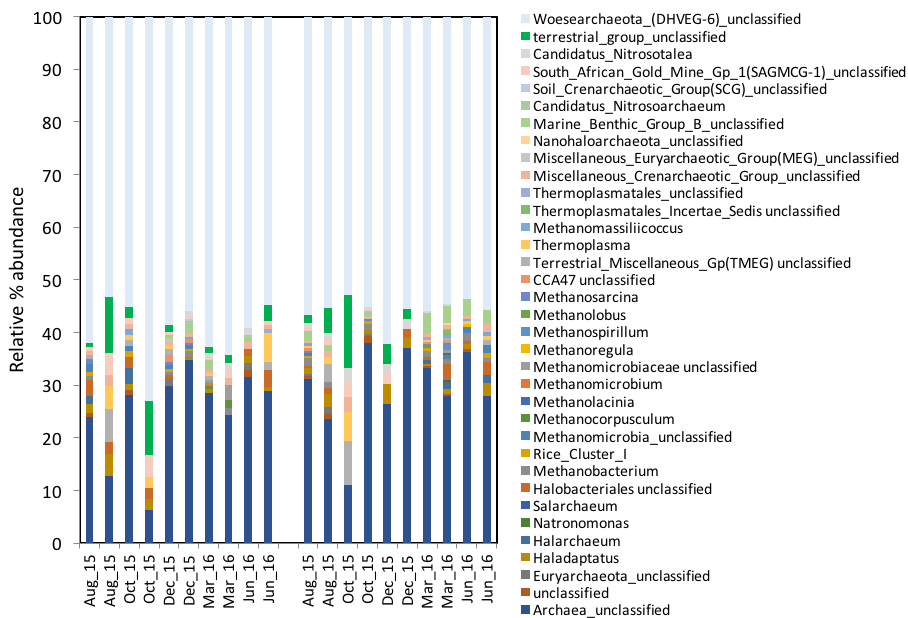


**Supplementary Figure S5:** Archaeal Genera community composition of surface sediment for the high elevation area (left) and low elevation area (right).

**Supplementary Table S1**: Alpha diversity statistics for microbial community OTUs at the high elevation site.

|  | nseqs | sobs | Inv  simpson | Inv  simpson_lci | Inv  simpson_hci |
| --- | --- | --- | --- | --- | --- |
| Aug_15(1) | 11062 | 1102 | 134 | 128 | 140 |
| Aug_15(2) | 11062 | 720 | 66 | 63 | 68 |
| Oct_15(1) | 11062 | 1003 | 139 | 134 | 144 |
| Oct_15(2) | 11062 | 637 | 67 | 64 | 70 |
| Dec_15(1) | 11062 | 1273 | 87 | 82 | 93 |
| Dec_15(2) | 11062 | 1526 | 149 | 142 | 156 |
| Mar_16(1) | 11062 | 1318 | 191 | 184 | 198 |
| Mar_16(2) | 11062 | 1094 | 109 | 104 | 113 |
| Jun_16(1) | 11062 | 895 | 70 | 67 | 74 |
| Jun_16(2) | 11062 | 1045 | 132 | 127 | 137 |
|  |  | **AVG** | **114** | **110** | **119** |

**Supplementary Table S2**: Alpha diversity statistics for microbial community OTUs at the low elevation site.

|  | nseqs | sobs | Inv  simpson | Inv  simpson_lci | Inv  simpson_hci |
| --- | --- | --- | --- | --- | --- |
| Aug_15(1) | 11062 | 1571 | 191 | 183 | 200 |
| Aug_15(2) | 11062 | 913 | 129 | 125 | 134 |
| Oct_15(1) | 11062 | 475 | 52 | 50 | 54 |
| Oct_15(2) | 11062 | 1554 | 183 | 175 | 193 |
| Dec_15(1) | 11062 | 844 | 96 | 92 | 100 |
| Dec_15(2) | 11062 | 1023 | 131 | 126 | 136 |
| Mar_16(1) | 11062 | 2024 | 214 | 202 | 228 |
| Mar_16(2) | 11062 | 2148 | 260 | 247 | 275 |
| Jun_16(1) | 11062 | 1174 | 101 | 97 | 106 |
| Jun_16(2) | 11062 | 761 | 35 | 33 | 36 |
|  |  | **AVG** | **139** | **133** | **146** |
